# Fluctuations of Attention and Working Memory

**DOI:** 10.5334/joc.70

**Published:** 2019-08-08

**Authors:** Kirsten C.S. Adam, Megan T. deBettencourt

**Affiliations:** 1 Department of Psychology, University of California San Diego, US; 2 Institute for Neural Computation, University of California San Diego, US; 3 Institute for Mind and Biology, University of Chicago, US; 4 Department of Psychology, University of Chicago, US

**Keywords:** Attention, Working memory, Short-term memory

## Abstract

Attention and working memory are intricately related, yet there remain ambiguities in how to best characterize this relationship. In his review, Oberauer formalizes several dimensions for the relationship between attention and working memory, focusing especially on the supporting role of attention during working memory maintenance. In this commentary, we highlight how attention and working memory relate on a broader time scale via trial-to-trial fluctuations. Specifically, we briefly describe evidence and implications of these fluctuations of attention and working memory. A strong link has been shown behaviorally (e.g., interleaved sustained attention and working memory tasks) and neurally (e.g., pre-trial predictors of working memory success), yet fluctuations of attention and working memory are also distinct. Thus, we argue that attention and working memory fluctuate synchronously but not synonymously.

Attention and working memory are fundamentally linked. Just as we can attend only a subset of perceptual information from the environment, we can actively maintain only a subset of this information in mind. The nature of this information or capacity limit, and whether it arises from attention, working memory, or a common resource, has been a source of ongoing debate. In his conceptual analysis and review in this issue, Oberauer (2019) delineates several axes by which we might define the relationship between attention and working memory and examines the literature for evidence of these potential definitions. He addresses how attentional constraints may generate working memory capacity limits during encoding and maintenance, focusing primarily on aggregate effects of attention and working memory (e.g., how average working memory performance differs with the addition of a secondary attention task). Although these aggregate measures are extremely important for understanding the overall relationship between attention and working memory, they disregard information about moment-by-moment fluctuations. In fact, an aggregate relationship between two cognitive processes does not require a moment-by-moment relationship. Here, we briefly describe evidence that both attention and working memory fluctuate over time. We propose that fluctuations of sustained attention represent a unique component of the relationship between attention and working memory and have profound consequences for what we remember.

Attention is not perfect, and we sometimes make errors even for the most trivial tasks, both in everyday life (Reason, 1984) and in the laboratory (Wolfe, Horowitz, & Kenner, 2005). These behavioral lapses are thought to be the consequence of exceptionally poor attentional states along a continuum. There is a long history examining fluctuations in our ability to detect stimuli, both faint (Guilford, 1927) and rare (Mackworth, 1948). Neural correlates of attention fluctuations have been observed using a variety of methodologies including EEG, fMRI, pupillometry, and population activity (Cohen & Maunsell, 2011; Ergenoglu et al., 2004; Esterman, Noonan, Rosenberg, & DeGutis, 2013; Esterman, Rosenberg, & Noonan, 2014; Unsworth, Robison, & Miller, 2018). Recent work has leveraged these behavioral and neural markers to track attention fluctuations over time and anticipate lapses (deBettencourt, Cohen, Lee, Norman, & Turk-Browne, 2015; deBettencourt, Norman, & Turk-Browne, 2018; Mathewson, Gratton, Fabiani, Beck, & Ro, 2009; Rosenberg, Finn, Constable, & Chun, 2015). Importantly, these sustained attention fluctuations are thought to be related to, but distinct from, our ability to transiently select, shift, and disengage attention (Chun, Golomb, & Turk-Browne, 2011; Esterman & Rothlein, 2019).

There is increasing evidence that moment-to-moment attentional state profoundly impacts memory (for review see Aly & Turk-Browne, 2017). Even prior to encoding a stimulus, hypothesized neural correlates of attention predict working memory performance. For example, higher pre-trial frontal theta power (Adam, Mance, Fukuda, & Vogel, 2015; Adam, Robison, & Vogel, 2018) and larger attention-directing ERPs (Murray, Nobre, & Stokes, 2011) have been associated with better working memory performance. In sum, ample evidence suggests that both working memory and attention performance fluctuate over time, and some evidence suggests that fluctuations of working memory performance may be driven by fluctuations of attentional state. Yet, one challenge is that working memory and attention fluctuations have typically been studied with separate tasks. To bridge these disparate paradigms, a new study interleaved two independent tasks (deBettencourt, Keene, Awh, & Vogel, 2019) to near-simultaneously monitor fluctuations of attention and working memory. Attention fluctuations were measured behaviorally via responses to a continuous performance task, and working memory was strategically probed during moments when attention was high or low (as indexed by ongoing performance in the attention task). This study revealed that attention fluctuations coincide with working memory performance fluctuations. Similarly, response fluctuations in an attention task also have long-term consequences on what is later remembered (deBettencourt et al., 2018).

Although there is some evidence that working memory and attention fluctuate together, it is also important to consider when they diverge. On the one hand, attention and working memory generally fluctuate in tandem (e.g., a better attentional state correlates with better memory). On the other hand, fluctuations of attention and working memory have strongly diverging time courses. A definitional hallmark of sustained attention tasks is a “vigilance decrement”, whereby performance worsens over time (e.g., Esterman, Reagan, Liu, Turner, & DeGutis, 2014). In contrast, working memory performance is surprisingly robust over time: Average performance stays constant even after hundreds of trials, or over an hour of the task (Adam et al., 2015). Likewise, attention fluctuations correlate with the number of items held in working memory but not the precision of a single representation (deBettencourt et al., 2019). In addition, sustaining spatial attention over a delay recruits different neural mechanisms than working memory (Hakim, Adam, Gunseli, Awh, & Vogel, 2019; Sheremata, Somers, & Shomstein, 2018). These neural discrepancies between fluctuations of attention and working memory offer a promising route to dissect their complex relationship. Future studies are needed to further delineate when attention and memory covary and, equally importantly, when they do not.

In sum, most work interrogating the relationship between attention and working memory has focused on their aggregate relationship, particularly during working memory encoding and maintenance. Here, we suggest that trial-to-trial attention fluctuations over time are also critical for understanding the intricate relationship between attention and working memory. Fluctuations of working memory and attention are highly coincident, but more work is needed to understand when and why working memory is affected by an optimal or suboptimal attentional state. Of course, “attention” is not a monolithic construct but a collection of mechanisms that enhance the representation of a subset of information. Similarly, attention fluctuations are likely multifaceted, and could influence working memory at different moments and by different means. For example, memory failures could arise because we fail to prepare for, apprehend, individuate, maintain, or retrieve information; fluctuations of attention could globally affect any or all of these sub-components of successful working memory performance. To fully characterize these cognitive processes, we should consider the moment-by-moment relationship between attention and working memory.
